# Hexagonal GaN nanorod-based photonic crystal slab as simultaneous yellow broadband reflector and blue emitter for phosphor-conversion white light emitting devices

**DOI:** 10.1038/s41598-019-55684-9

**Published:** 2020-01-15

**Authors:** Suk-Min Ko, Joonseok Hur, Chulwon Lee, Isnaeni, Su-Hyun Gong, MinKwan Kim, Yong-Hoon Cho

**Affiliations:** 1 Department of Physics, Korea Advanced Institute of Science and Technology (KAIST), Daejeon, 34141 Republic of Korea; 2 Graduate School of Nanoscience and Technology, Korea Advanced Institute of Science and Technology (KAIST), Daejeon, 34141 Republic of Korea; 3 KI for the NanoCentury, Korea Advanced Institute of Science and Technology (KAIST), Daejeon, 34141 Republic of Korea; 4 Present Address: Department of Physics and Research Laboratory of Electronics, Massachusetts Institute of Technology, Cambridge, Massachusetts 02139 United States

**Keywords:** Optics and photonics, Applied physics, Condensed-matter physics, Optical physics

## Abstract

We report a hexagonal GaN nanorod-based two-dimensional photonic crystal (PhC) slab for phosphor-conversion white light emitting devices analyzed by three-dimensional finite-difference time-domain simulation; this slab has a broad reflection band at yellow wavelength with low Fabry-Pérot background at normal incidence. For practical use as a wavelength-selective reflector, a buffer layer under the PhC slab is employed to sustain the nanorods in the PhC slab. However, we observed that the buffer layer placed below the slab destroys the broad reflection band due to evanescent coupling of electromagnetic field in the slab and the buffer layer. By introducing small-sized base pillars between the slab and the buffer layer, we could decouple the interaction between the slab and the buffer layer and maintain the broad reflection band without any unexpected dips. Since this GaN nanorod-based PhC slab is designed for practical light emitting devices by considering dielectric and transparent conducting layers, this structure is directly applicable for developing hybrid white light emitting devices having both an (active) blue-color-emitting nanorod emitters and a (passive) normal reflector of phosphor emission.

## Introduction

Recently, there has been a growing interest in GaN nanorods with GaN/InGaN axial or core-shell heterostructures as a form of periodic array for various photonic applications such as light emitting diodes (LEDs) and solar cells due to their superior emission efficiency as well as mechanical and chemical stabilities. This periodic GaN nanorod array is able to act as an in-plane photonic crystal (PhC) structure or a PhC slab for normal direction by controlling the size and the arrangement of rods. Two-dimensional (2D) PhC slab has been extensively studied due to its substantially high reflectivity and compactness at normal incidence^1,2^. This high reflectivity of the 2D PhC slab originates from guided-mode resonance, which occurs when a normally incident wave is coupled with slow Bloch modes above the light line in the photonic band structure of the slab. The spectral shape of the resonant peak due to the guided-mode resonance can be engineered by adjusting the structural parameters of the PhC slab, such as the periodicity, slab thickness, and index contrast depending on their purpose, e.g. high-quality factor (Q) narrow band filters or low-Q broadband reflectors. There were several studies to determine the origin of these resonant peaks of 2D PhC slab and to apply these structures to sensors that can use the high-Q resonant peaks^3,4^. Also, it was theoretically and experimentally observed that a single layer of a dielectric 2D PhC slab with an air hole array can have a low-Q wide reflection band that is far more compact than a traditional 1D distributed Bragg reflector^5–9^.

In this work, we investigated the 2D PhC slab composed of GaN nanorod array to establish a broadband reflector which specifically provides high and broad reflection at yellow wavelength region and high transmission at blue wavelength region. This feature can be utilized for improving the efficiency of phosphor-conversion white LEDs consisting of a blue LED and a yellow phosphor. Here, their reflection and transmission properties were systematically studied by using three-dimensional (3D) finite-difference time-domain (FDTD) simulator in order to use the GaN-based nanorod array as a normal broadband reflector of yellow phosphor emission while transmitting blue color emission from a blue LED. Moreover, it is worth noting that the GaN-based nanorod array itself can be a blue-color-emitting nanorod LED having InGaN-based active layer(s), such as InGaN/GaN axial and/or core-shell heterostructures, in the nanorods. The rod-based structure was chosen because it is preferable to make low Fabry-Pérot background as it generally has much lower filling fraction than conventional hole array in dielectric layer. This wavelength-selective reflection property and the textured structure of our GaN nanorod-based PhC slab can simultaneously improve the color rendering and extraction efficiency of the phosphor-conversion white LEDs by enhancing the reflection of phosphor emission, in contrast to the previously proposed hole array on GaN-based LEDs which were used to enhance only the extraction efficiency of the LEDs^10,11^. Moreover, we calculated the reflection spectra of the nanorod-based PhC slab with a GaN buffer layer, a GaN base pillar, a sapphire substrate, a mask material needed for the nanorod formation, and thin transparent conducting layer (TCL) [e.g., indium tin oxide (ITO)] in consideration of the actual device fabrication and electrical operation. The destruction of reflection band by the buffer layer via evanescent coupling was numerically observed, and therefore, introduction of the base pillar array between the PhC and the buffer layer was suggested to prevent this kind of coupling. Lastly, we confirmed that the introduction of a sapphire substrate, a mask material, and ITO do not significantly affect to the broad reflection band.

## Results

### Simulation model and method

**Figure 1:**
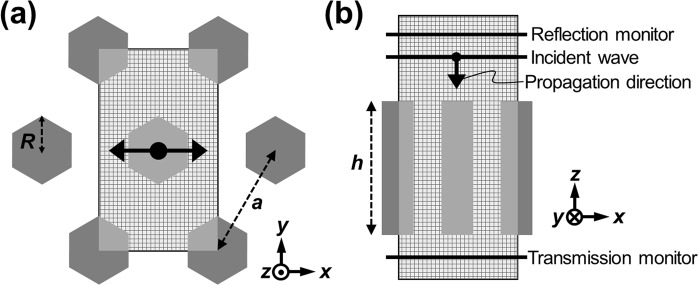
Schematic illustrations of the computational domains of GaN nanorod-based PhC slab: (**a**) top view and (**b**) cross-sectional view of the structure.

**Figure 2:**
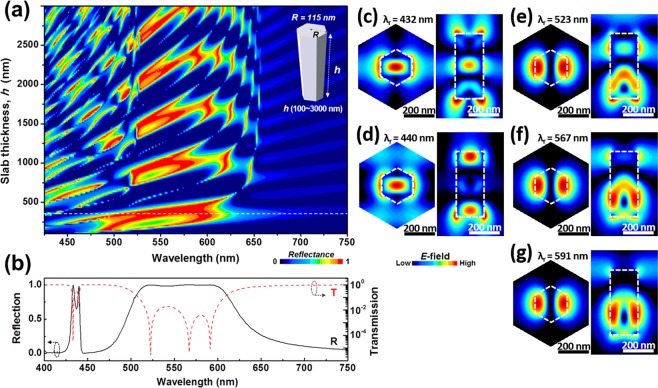
(**a**) Reflection map of nanorod-based PhC slab plotted versus wavelength and *h* with fixed *a* = 480 nm and *R* = 115 nm. (**b**) Reflection (black solid line) and transmission (red dashed line) spectra of the nanorod-based PhC slab at an optimized condition (*h* = 365 nm). (**c–g**) Electric field distributions across *xy*- and *xz*-planes at five resonant peaks λ_r_ = (**c**) 432, (d) 440, (**e**) 523, (**f**) 567, and (**g**) 591 nm in (**b**). The positions of *xy*-planes in *z*-direction are −5, 150, 125, 99 and 83 nm, respectively. The distribution in *xy*-plane is shown in the Wigner-Seitz cell of the PhC.

Figure 1 presents the computational configurations of our GaN nanorod-based PhC slab. The GaN nanorod is designed to have the hexagonal shape in consideration of the (bottom-up) growth of its wurtzite crystal structure. The structure has a lattice constant (*a*), slab thickness (*h*), refractive index (~2.4 for GaN), and radius (*R*) for the GaN nanorod. The computational domain is enclosed by Bloch boundary conditions on the four sidewalls and by perfectly matched layers on the top and bottom displaced from top and bottom of PhC slab by 2100 nm and 1400 nm, respectively. A plane wave source is placed 700 nm above the slab to propagate along the normal direction of the slab, as shown in Fig. 1(b). The spectral analysis of these structures was performed by a commercial FDTD simulation tool (FDTD solutions, Lumerical solutions, Canada). The transmitted and reflected waves were detected by the monitors positioned 700 nm below and 1400 nm above the PhC slab, respectively. The source plane, reflection monitor, and top boundary of the simulation domain are pushed further up when there is a buffer layer, a gap or base pillars, or a sapphire substrate by the total thickness of the additional structures. Adding thin SiO2 masks and ITO coatings in Fig. 2 does not change the positions of the objects. Non-uniform mesh adapted to the distribution of the refractive index is used to minimize numerical dispersion^12–14^. The mesh size used in the calculation was 6.5 nm in GaN nanorod structure and smaller than one 22nd of wavelength over the computation domain and the wavelength range of interest (400–750 nm).

**Figure 3:**
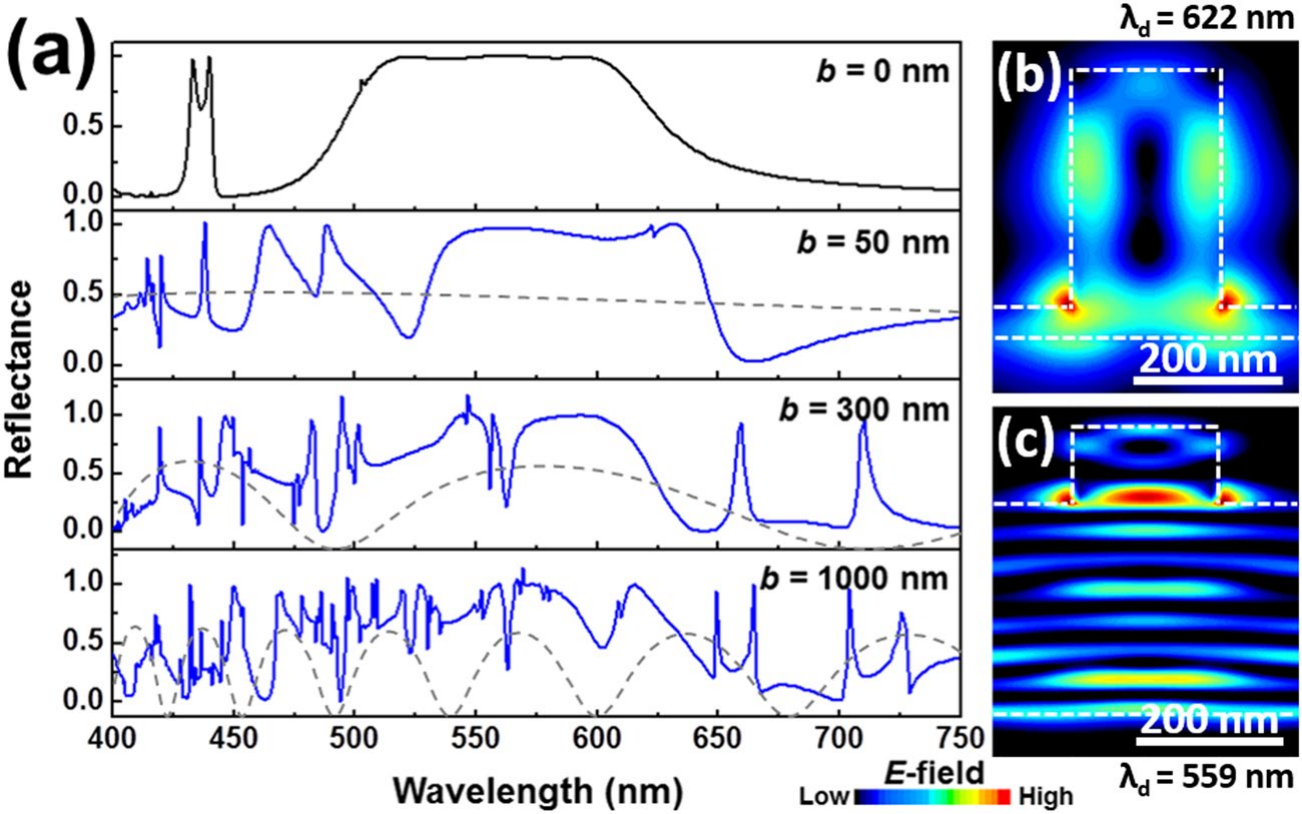
(**a**) Reflection spectra of the nanorod-based PhC slab (*a* = 480 nm, *R* = 115 nm, and *h* = 365 nm) on a buffer layer with various *b*. Grey dashed lines show Fabry–Pérot reflection spectra of buffer layers only. Electric field distributions at (**b**) *λ*_d_ = 622 nm (*b* = 50 nm), and (**c**) *λ*_d_ = 559 nm (*b* = 1000 nm).

**Figure 4:**
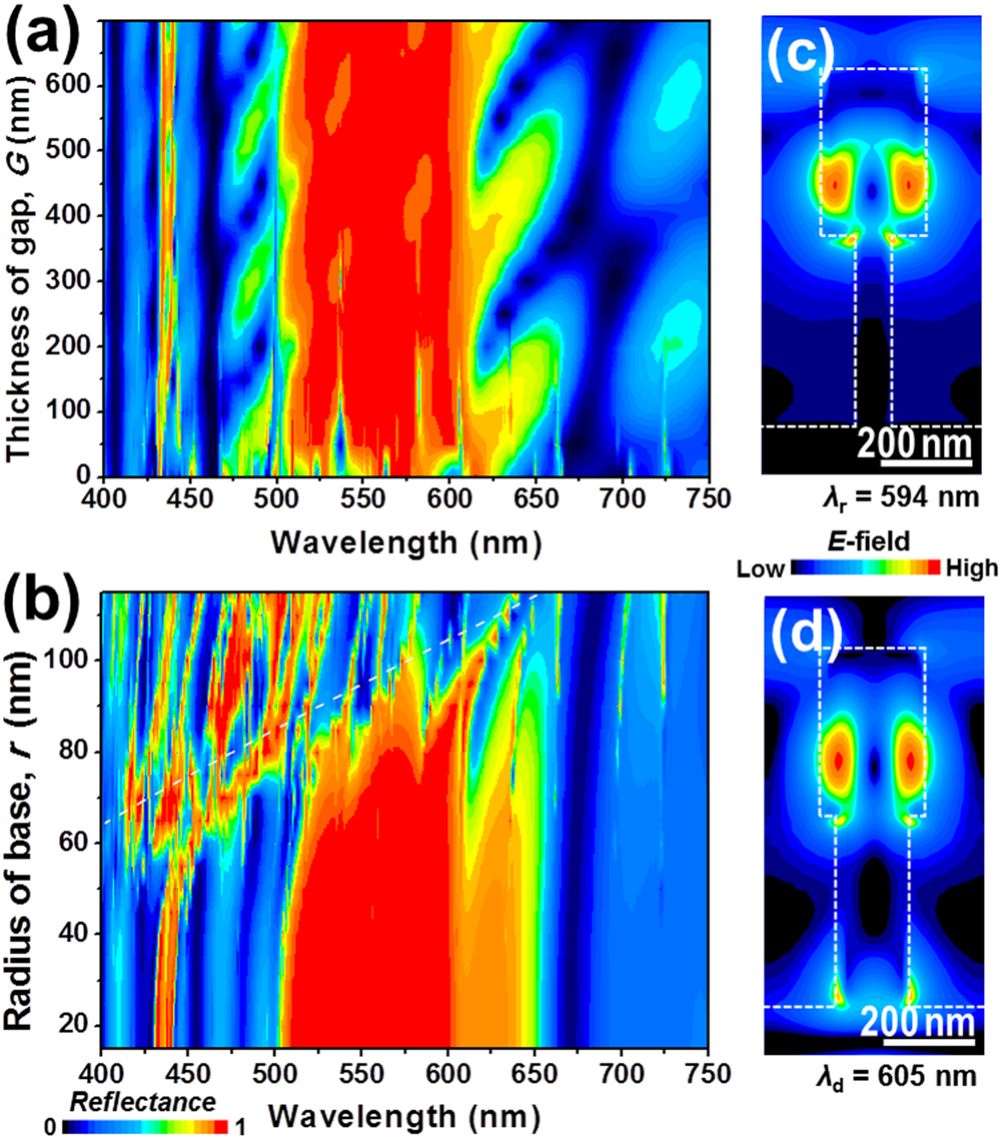
(**a**) Evolution of the reflection spectra as a function of *G* between the PhC slab (*a* = 480 nm, *R* = 115 nm, and *h* = 365 nm) and the buffer layer (*b* = 1000 nm). (**b**) Reflection spectra of the structure with different *r* from 15 nm to 115 nm. White dashed line shows cutoff wavelength versus radius of dielectric cylinder given in Eq. (1). Electric field distributions with (**c**) *r* = 40 nm (at *λ*_r_ = 594 nm) and (**d**) *r* = 80 nm (at *λ*_d_ = 605 nm).

### Fano-shaped guided-mode resonance and effect of slab thickness

In general, it is well known that the spectral linewidth of a resonant peak is proportional to the loss of the leaky waveguide mode, which is affected by *h*, *a*, and *R*^1,7^. We investigated the variation in the reflection spectra at normal incidence with varying *h* for fixed values of *a* and *R* of the nanorod-based PhC slab to find the widest reflection band in yellow wavelength region. Figure 2(a) presents the reflection map of the nanorod-based PhC slab plotted versus wavelength and *h* with *a* = 480 nm and *R* = 115 nm. There are a great number of modal shaped features in the reflection map, as shown in Fig. 2(a). Some of the modes create S-shaped high reflection bands through overlapping with neighboring modes at a certain *h*, while others are isolated without the interaction. The *S*-shaped broad reflection bands at a certain *h* have high reflectivity because the *h* of the PhC slab is well matched with the boundary conditions for the normally incident light to efficiently excite the leaky modes of the PhC slab by forming a standing wave, despite of their low resonant peak efficiency due to the low Q^7^. Interestingly, above the 655 nm wavelength region in the reflection map there are no modal features but only a Fabry-Pérot background. It is because the electric field of wavelength longer than the cutoff wavelength (*λ*_cutoff_) of an individual nanorod in the slab exhibits single mode only. *λ*_cutoff_ is given by:

1 $${{\rm{\lambda }}}_{{\rm{cutoff}}}=\frac{2\pi R}{V}\sqrt{n{({{\rm{\lambda }}}_{{\rm{cutoff}}})}^{2}-1},$$

where normalized frequency *V* is 2.4048 for dielectric cylinder of radius *R* and *n* is ordinary refractive index for GaN at 300 K given by ref. ^15^. Calculated *λ*_cutoff_ by solving Eq. (1) for *R* = 115 nm was 649.1 nm, agreeing with the FDTD results (655 nm) with an error less than 1%. As a result, we obtain the widest reflection band (from 515 to 600 nm) and low Fabry-Pérot background (>650 nm and ~450 nm) with *h* = 365 nm at which condition forms a first-order standing wave inside the PhC slab as shown in Fig. 2(a) by dashed line. As the black solid line in Fig. 2(b) indicates, the broadest reflection band of over 99.2% in the yellow wavelength region (from 515 to 600 nm) and low Fabry-Pérot background (<1%) in the blue wavelength region (~450 nm) are formed under the optimized simulation conditions. The logarithmic scale plot of the transmission spectrum (red dashed line) clearly shows that this reflection band consists of the three resonant peaks of 523, 567, and 591 nm generated by the radiative coupling between the incoming wave and the leaky waveguide modes of the PhC slab, referred as guided-mode resonance. The five resonant peaks exhibit over the spectrum range from 400 to 750 nm, which means that the five leaky modes are excited by the incident wave in the Γ-point of the dispersion band diagram of the nanorod-based PhC slab^1,6^. We also compared the resonant peaks with photonic band structure excited by plane wave, to provide more comprehensive explanation on the leaky nature of those five leaky modes. The calculated band diagrams can be found in the Supplementary Material (Fig. S1). Moreover, we confirmed that the reflection spectral shape and the positions of the resonant peaks were not influenced by the polarization angle of the normal incident wave due to the 60° rotational and translational symmetries of nanorod-based PhC slab structure^6,9,16^. Because the incident waves to the nanorod-based PhC slab can be the sum of several waves with various polarization angles in real situations, this polarization angle-independent property of our nanorod-based PhC slab is one of its great merits as a reflector. The guided-mode resonance-induced wideband reflection of periodic structures of finite thickness has been extensively studied both theoretically^7,17–19^ and experimentally^16,20–22^, for 1D and 2D PhC slab. In particular, the modal shape of the reflection over increasing thickness in Fig. 2(a) and the broad reflection band with the resonant transmission dips in Fig. 2(b) are very similar to those of 1D PhC in ref. ^7^.

**Figure 5:**
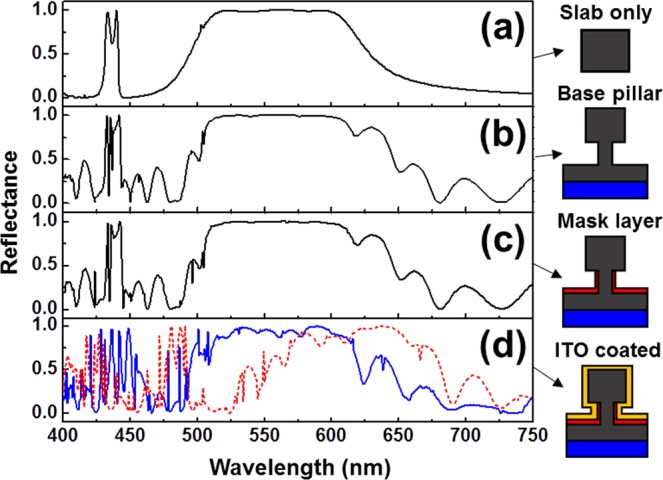
Reflection spectra with various GaN nanorod-based PhC slab structures: (**a**) nanorod-based PhC slab (*a* = 480 nm, *R* = 115 nm, and *h* = 365 nm) only, (**b**) the PhC slab with GaN base pillars (*r* = 45 nm and *G* = 400 nm), (**c**) the PhC slab with GaN base pillars and 10-nm-thick SiO_2_ mask, and (**d**) the PhC slab with GaN base pillars and SiO_2_ mask coated by 25-nm-thick ITO layer for current injection, showing a redshift of resonant peaks (red dotted line). By reducing *R* and *h* in the PhC slab (*R* = 80 nm and *h* = 310 nm), the widest reflection band of 96.4% in the yellow wavelength region is obtained (blue solid line). All the PhC slabs with GaN base pillars in (**b**–**d**) are formed on a 1-μm-thick GaN buffer layer and a 2-μm-thick sapphire substrate.

### Effect of GaN buffer layer below the slab

Investigation of the effect on the reflection spectra of the GaN nanorod-based PhC slab by introducing a GaN buffer layer below the slab is necessary because the buffer layer below the slab is essential to sustain the nanorod array for practical device applications. (For instance, the GaN nanorod array can be formed on a GaN layer as a result of the lithography and top-down etching processes.) Fig. 3(a) shows the reflection spectra of the GaN nanorod-based PhC slab (*a* = 480 nm, *R* = 115 nm, and *h* = 365 nm) on a GaN buffer layer with various buffer thicknesses (*b*). By employing the buffer layer below the PhC slab, high-Q dips start to be progressively formed within the broad reflection region. These high-Q dips originate from the low radiative coupling of the modes with long lifetime inside the buffer layer in a form of the film, interacting with the PhC slab. In general, the guided modes of a homogeneous film are not coupled to radiation modes due to its continuous translational symmetry^1^. However, the guided modes of the buffer layer can be excited by coupling from the modes of the PhC slab, forming additional high-Q dips in the reflection spectra. This effect was directly confirmed by observing field distributions of PhC slab on a buffer layer: Figs. 3(c–g), 2(g) show the electric field distributions in PhC slabs without a buffer layer, with the buffer of thickness b = 50 nm and 1000 nm, respectively. For the PhC slab without a buffer layer, the mode concentrates only in and at the surface of the slab at three resonance wavelengths in the broad reflection band [Fig. 2(e–g)], while the electric field in the PhC slab with a buffer layer simultaneously exists in the PhC slab and the buffer layer due to the coupling between them [Fig. 3(b,c)].

### Decoupling of resonances by employing gap or base pillars between slab and buffer layer

In order to keep the broad reflection band feature without additional high-Q resonant dips, we investigate the critical gap thickness between the nanorod-based PhC slab and the buffer layer, in which the evanescent field from the slab does not reach the buffer layer. Figure 4(a) shows the evolution of the reflection spectra as a function of gap thickness (*G*) between the PhC slab (*a* = 480 nm, *R* = 115 nm, and *h* = 365 nm) and the buffer layer (*b* = 1000 nm). We found that additional high-Q resonant dips caused by the excitation of the modes in the GaN buffer layer start to disappear when the slab is 200 nm away from the GaN buffer layer. This is due to the presence of less evanescent coupling from the PhC slab into the buffer layer. Because the evanescent field from the slab decays within a half-wavelength scale, the broad reflection band in the reflection spectrum fully recovers when *G* = 400 nm. Moreover, we introduced GaN base pillars into the gap between the PhC slab and the buffer layer to support the GaN nanorod array. We emphasize that this supporting pillars are essential in practice and can be realized by using the selective epitaxial growth method, in which the GaN nanorod array can be grown on a GaN buffer layer with a dielectric mask having opening array between GaN nanorod array and the buffer layer. GaN would be initially grown through the opening array area of the dielectric mask (i.e., base pillars) and then form GaN nanorod array with a certain level of lateral growth (i.e., nanorod PhC slab). After the growth, the dielectric mask can be easily removed by chemical etching method, forming the base pillars surrounding air gaps. To find the optimized radius of the base pillars, a reflection map of the structure (*a* = 480 nm, *R* = 115 nm, *h* = 365 nm, *b* = 1000 nm, and *G* = 400 nm) with different values of base pillar radius (*r*) from 15 nm to 115 nm was calculated as shown in Fig. 4(b). While the reflection band is maintained when *r* < 50 nm, it significantly shrinks as *r* increases from 50 to 100 nm. It is because an increase of *λ*_cutoff_ of the base pillars, which is nearly proportional to *r* as the dashed line in Fig. 4(b), allows the propagation of electric field from the PhC slab into the buffer layer through the base pillars, resulting in the formation of high-Q dips. The excitation of modes in the buffer layer through the base pillar is directly confirmed in Fig. 4(c,d) when *r* = 40 nm (at *λ*_r_ = 594 nm) and *r* = 80 nm (at *λ*_d_ = 605 nm), respectively. When *r* = 40 nm, most of the electric field exists only in the nanorod-based PhC slab at the resonance, whereas the field is not only in the PhC slab but also located inside the base pillar and in the buffer layer when *r* = 80 nm due to the propagation of electric field from the PhC slab through the thick base pillar.

### Effect of additional layers required for practical LED fabrication

Finally, the effects of additional components, which are necessary for actual device application, on the reflection spectrum of nanorod-based PhC slab were investigated. Figure 5 shows the reflection spectra with various structures such as (a) GaN nanorod-based PhC slab only, (b) the PhC slab with GaN base pillars (*r* = 45 nm and *G* = 400 nm) [together with a GaN buffer layer (1 μm) and a sapphire substrate (2 μm) for (b) to (d) cases], (c) the PhC slab with GaN base pillars and thin dielectric (SiO_2_) mask layer, and (d) the PhC slab with GaN base pillars and thin dielectric mask layer, followed by TCL (ITO) coating for current injection purpose. To site-selectively fabricate the PhC slab and the base pillars with a fixed radius, length, and lattice constant, the nano-patterned dielectric mask is required. We found that the reflection spectral shape is not influenced by introducing the thin (10 nm) SiO_2_ mask. Lastly, for current injection we employed the 25 nm thick ITO layer (refractive index: ~2) on the PhC slab and dielectric mask layer. As the red dotted line in Fig. 5(d) indicated, there is a redshift of resonant peaks by adding the ITO layer on the structure due to the increase of effective refractive index of the structure. After slightly reducing the radius and length of the rod in the PhC slab (*R* = 80 nm and *h* = 310 nm), we achieved the widest reflection band of 96.4% in the yellow wavelength region (from 515 to 600 nm) of the nanorod-based PhC slab covered with the ITO layer, as the blue solid line in Fig. 5(d) presented.

## Discussion

For the practical applications, based on our simulation results, the GaN nanorod-based PhC slab structures can be utilized in two different ways: (i) simultaneous broad yellow band reflector and blue band transmitter (with a separate blue LED) or (ii) simultaneous broad yellow band reflector and blue light emitter itself. For the case (i) which acts as a passive multi-functional filter, either the PhC slab only possibly with a low index matrix [Fig. 5(a)] or the PhC slab with base pillars on a buffer layer with a substrate [Fig. 5(b)] can be placed between a phosphor layer (on top) and a blue LEDs (at the bottom). For the case (ii) which functions as a passive-active hybrid device, the PhC slab with base pillars (without or with a thin dielectric layer) [Fig. 5(b,c)] or the PhC slab with base pillars and a thin dielectric layer, followed by a TCL coating [Fig. 5(d)] can be directly used with a phosphor layer on top of this active device and operated by optical pumping or current injection, respectively.

We note that the dielectric layer with opening area is required for site-selective growth of the nanorod PhC slab and base pillars by the selective epitaxial growth and can be partially or fully removed by simple etching method. The thin dielectric layer can be still needed for protecting leakage current and solely opening the p-type layer of the GaN nanorods and the TCL is used for better current injection through the opened p-type layer of GaN nanorods. In case of core-shell (axial) type heterostructures or quantum well structures on the nanorods, the p-type layer is typically grown on the surface (top) of GaN nanorods. Therefore, for practical device fabrication and electrical operation, we expect that employing the GaN base pillars, the low refractive index dielectric thin mask layer, and the TCL for current injection will be a terrific way to maintain the high- and broad-reflection band of the GaN nanorod-based PhC slab at normal incidence.

## Conclusion

Using the 3D FDTD method, we investigated a hexagonal GaN nanorod-based 2D PhC slab with a triangular lattice for phosphor-conversion white light emitting device applications. We obtained the high reflection band in yellow wavelength region originated from guided-mode resonance and a low Fabry-Pérot background in blue wavelength region due to low material filling factor of nanorod array with optimized parameters. A buffer layer is added under the PhC slab to sustain the nanorods in the PhC slab, but we found that the buffer layer destroys the broad reflection band due to evanescent coupling between the slab and the buffer layer. By further employing the GaN base pillars between the slab and the buffer layer, we were able to decouple the interaction between the slab and the buffer layer and achieve good wavelength-selective properties from the structure at normal incidence without any unexpected dips. The radius of the base pillars should be sufficiently small to prevent the generation of the leaky modes inside the base pillar in consideration of evanescent coupling length and cutoff wavelength. For the practical use, we also combined this GaN nanorod-based PhC slab structures with dielectric and transparent conducting layers and found that the high- and broad-reflection band feature at normal incidence was maintained. Therefore, this GaN nanorod-based PhC slab structure have very unique feature for developing hybrid white light emitting devices acting as an (active) blue-color-emitting nanorod emitter and a (passive) normal reflector of phosphor emission simultaneously.

## Supplementary information

Supplementary Information
