# Supplementary material for: Hexagonal GaN nanorod-based photonic crystal slab as simultaneous yellow broadband reflector and blue emitter for phosphor-conversion white light emitting devices
Source: Sci Rep. 2020 Jan 15;10:358. doi: 10.1038/s41598-019-55684-9 (PMC6962447; doi:10.1038/s41598-019-55684-9)
Supplement: Supplementary file 1 — Supplementary Information [file 41598_2019_55684_MOESM1_ESM.pdf]

Supplementary Material:

Hexagonal GaN nanorod-based photonic crystal slab as simultaneous yellow broadband reflector and blue emitter for phosphor-conversion white light emitting devices

Suk-Min Ko[1†], Joonseok Hur[1,4†], Chulwon Lee[1], Isnaeni[1], Su-Hyun Gong[1], Min Kwan Kim[2], and Yong-Hoon Cho[1,3*]

[1]Department of Physics, Korea Advanced Institute of Science and Technology (KAIST), Daejeon 34141, Republic of Korea

[2]Graduate School of Nanoscience and Technology, Korea Advanced Institute of Science and Technology (KAIST), Daejeon 34141, Republic of Korea

[3]KI for the NanoCentury, Korea Advanced Institute of Science and Technology (KAIST), Daejeon 34141, Republic of Korea

[4]Present address: Department of Physics and Research Laboratory of Electronics, Massachusetts Institute of Technology, Cambridge, Massachusetts 02139, United States.

†These authors contributed equally to this work.

* yhc@kaist.ac.kr

The band structures of 2D GaN hexagonal rod-based photonic crystal slab excited by plane wave at normal incident

The band structures of the photonic crystal (PhC) slab with optimized geometry for broad reflection band (a = 480 nm, R = 115 nm, and h = 365 nm) were calculated by adding time monitors, distributed at random positions within the PhC slab, to the simulation setup shown in Fig. 1. The symmetric/anti-symmetric condition along the z-direction with respect to the xy-plane across the center of the PhC slab is imposed to obtain the even/odd-mode band structure. The spectrum of electric field inside the PhC slab is calculated by taking the Fourier transform of the recorded field with the time monitors. The spectra over K vectors in the irreducible Brillouin zone are calculated by changing K_x and K_y of Bloch boundary condition in side walls. The plane wave at normal incidence was used as an illuminating source to investigate the relevant modes in the situation considered in the paper. The results are presented in Fig. S1.

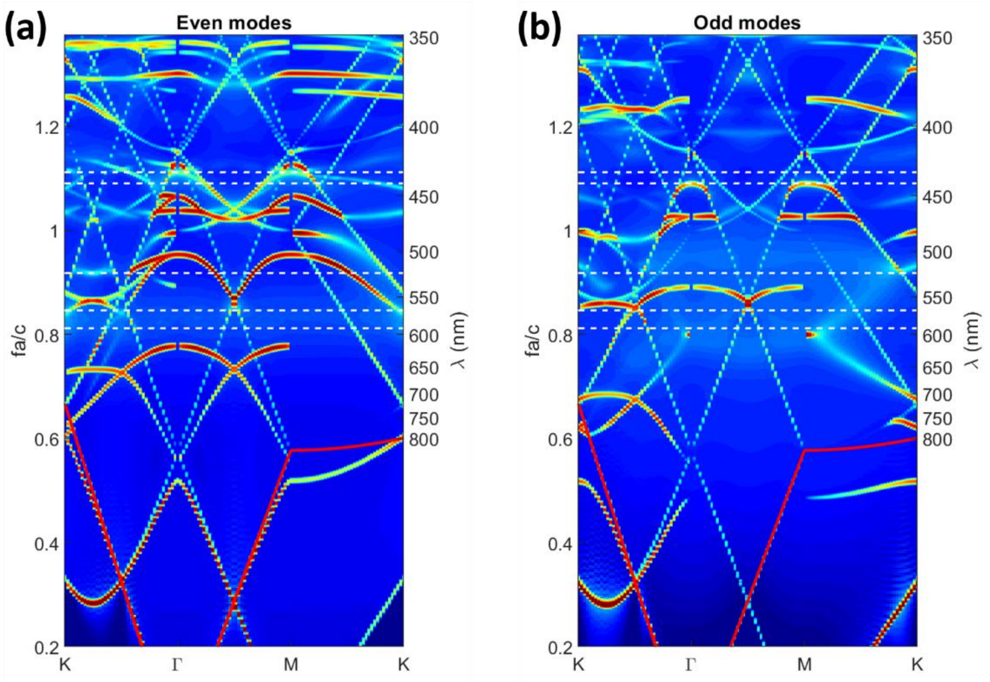

Figure S1. Calculated band structures excited by plane wave at normal incidence for even-mode (a) and odd-mode (b). Five horizontal white dashed lines correspond to the wavelengths of five transmission dips in Fig. 2(b).

The reflection band in manuscript figure 2(b), ranging from 500 to 600 nm can be explained by the absence of a significant photonic band either from even and odd mode photonic crystal band structure with plane wave excitation. Please note that there are somewhat leaky modes existing in this range as the transmission dips suggest. One may notice that there exists a very 'blurry' mode, which corresponds to the white dashed lines at around 560 and 600 nm. Those are well matched to the dips observed from transmission spectra. The modes are blurry due to its very low Q factors, as the wide linewidths of the dips in the transmission spectrum imply.

Likewise, the reflection band and the resonance dips at blue-range also can be explained. The two transmission dips in blue-range show relatively narrow linewidth, indicating the existence of higher Q modes. Those modes are clearly observed from the higher order Γ-point of even-mode band structure [Fig. S1(a)] and odd-mode band structure [Fig. S1(b)], indicated by white dashed lines, respectively.
